# Supplementary material for: Plasma Proteomic Analysis Reveals the Potential Role of Lectin and Alternative Complement Pathways in IgA Vasculitis Pathogenesis
Source: Diagnostics (Basel). 2023 May 12;13(10):1729. doi: 10.3390/diagnostics13101729 (PMC10217085; doi:10.3390/diagnostics13101729)
Supplement: Supplementary file 1 [file diagnostics-13-01729-s001.zip › diagnostics-2271109-supplementary.pdf]

Sample preparation

Blood samples were collected in EDTA-containing tubes and immediately centrifuged (10 min at 2000xg) at 4 °C. Plasma was removed and stored at 80 °C until analysis. Sample preparation was carried out using PreOmics İST Kit according to the manufacturer's protocol (PreOmics GmbH, Germany). Plasma tryptic digests were prepared by diluting plasma 1:10 with MS-grade deionized water prior to denaturation. Each sample was lysed with 50 µL of lyse buffer and denatured at 95 °C at 1000 rpm for 10 min. The denatured sample was mixed with 50 µL trypsin/LysC mixture solution and incubated for 3 h at 37 °C on heating block. The reaction was subsequently terminated with 100 µL stop solution and mixed thoroughly. Peptides were cleaned up by spinning the cartridge containing peptide digest 3 min at 6400 rpm followed by two washing steps and peptide elution. At this point, fractionation was performed by a two-step salt gradient (100 mM Ammonium formate, 40% ACN, 0.5% Formic acid, and 150 mM Ammonium formate, 60% ACN, 0.5% Formic acid) to increase the identified protein number. Final fractionation step was performed with the elution buffer provided by the PreOmics kit. Every elution pool was concentrated in vacuum concentrator at 45°C. Then, the peptides were dissolved in LC loading solution and sonicated for 5 min. The solutions were stored at -80°C until the measurements.

Nano liquid chromatography-tandem mass spectrometry (nLC-MS/MS)

Plasma digests were separated and measured by using nLC-MS/MS consisting of a nLC (Bruker nanoElute), combined with a QTOF mass spectrometer (Bruker Maxis II ETD) having nano-electrospray ion source (Captive Spray, Bruker). Cleaned-up peptides were separated on 15 cm nLC column [ID 75 µm; in-house packed into the tip with ReproSil C18, 1.9 µm, 120 Å resin (Dr. Maisch GmbH)] at 37 ºC. For each LC-MS/MS analysis, 1 µL digested plasma solution was used for 80-min runs. Peptides were loaded in 2% Acetonitrile/ 98% Water (0.1% (v/v) TFA) and eluted with a linear 40-min gradient of 2–17% of buffer B (0.1% (v/v) formic

acid, Acetonitrile), followed stepwise by a 20-min increase to 25% of buffer B, a 10 min to 40% of buffer B, 5-min increase to 80% of buffer B, followed by a 5-min wash of 80% buffer B at a flow rate of 400 nL/min. Column temperature was kept at 37°C by a Peltier element containing heater. MS data were acquired with a data-independent acquisition (DIA) method. The DIA-MS method consisted of an MS^1 scan from 350 to 2200 m/z range at a resolution around ~80,000, and, auto MS/MS mode was applied 150 to 2200 m/z mass range around same resolution via selection of active exclusion mode.

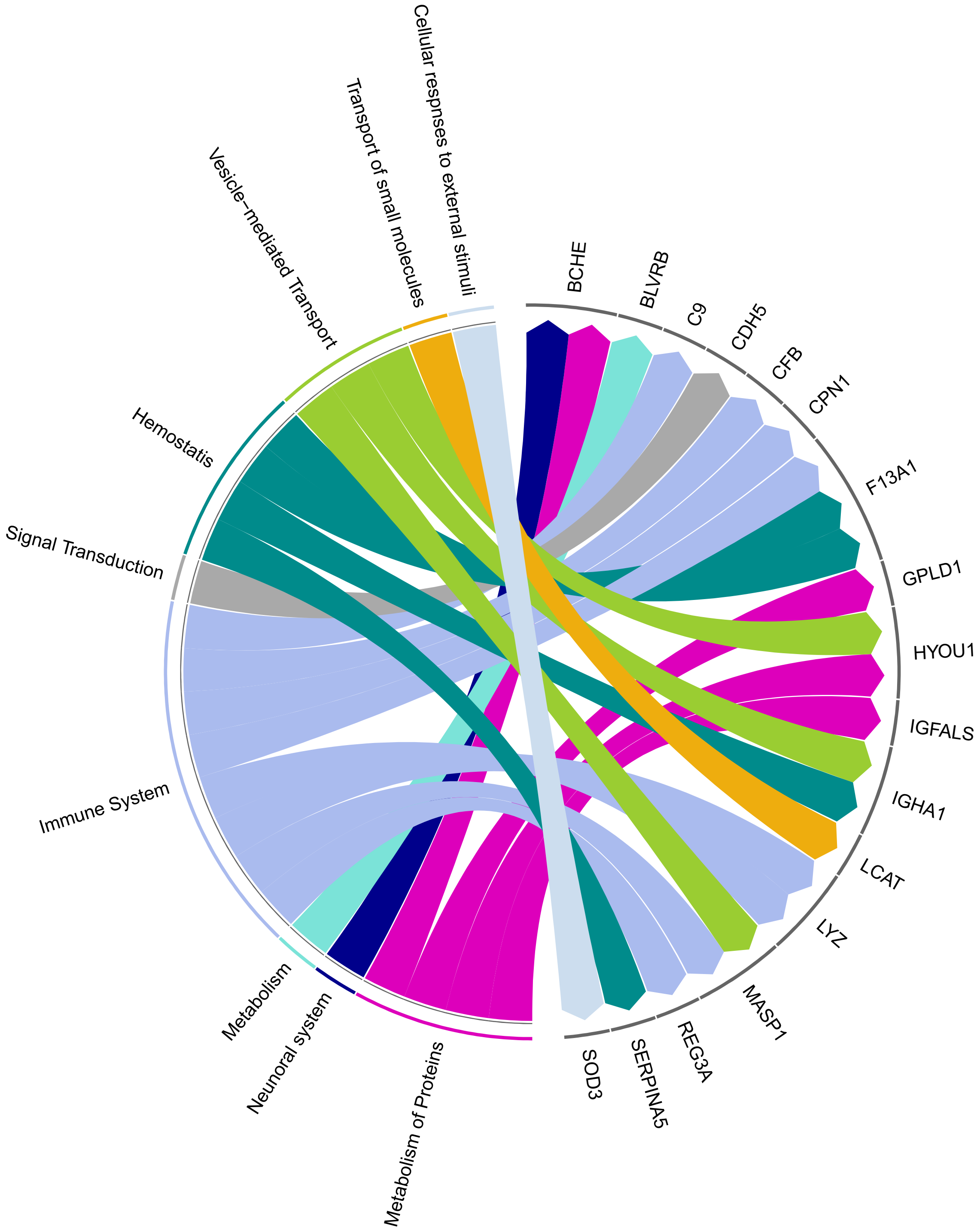

Supplement Figure S1. Reactome Pathway Analysis of the differentially expressed proteins in children with IgAV. Reactome database defined 10 different pathways for 17 out of 20 differentially expressed proteins.

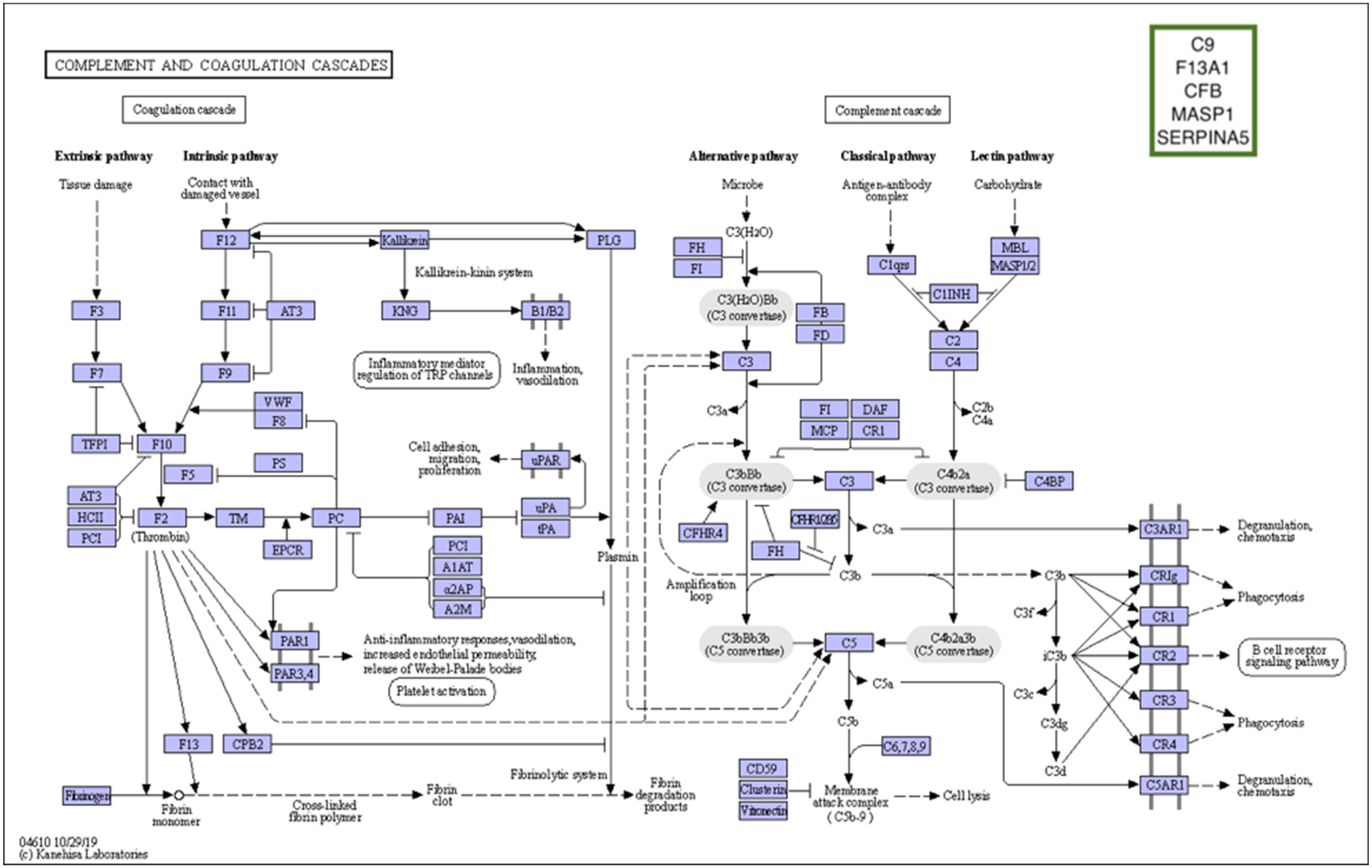

Supplement Figure S2. Complement and coagulation cascades pathway retrieved from KEGG.

Supplement Table S1. Gene Ontology results fort he significant proteins.

Functional Analysis	GO Term	#Genes	Gene Names	Panther Protein Class
Biological Process	biological adhesion (GO:0022610)	1	CDH5	cadherin
Biological Process	biological regulation (GO:0065007)	7	IGHA1<br>SERPINA7<br>SERPINA5<br>F13A1<br>REG3A<br>HYOU1<br>CDH5	immunoglobulin<br>protease inhibitor<br>protease inhibitor<br>transferase<br>-<br>-<br>cadherin

Biological Process	cellular process (GO:0009987)	9	CPN1<br>SERPINA7<br>SERPINA5<br>SOD3<br>DNAH3<br>F13A1<br>REG3A<br>HYOU1<br>CDH5	protease<br>protease inhibitor<br>protease inhibitor<br>oxidoreductase<br>microtubule binding motor protein<br>transferase<br><br>-<br>-<br>cadherin
Biological Process	developmental process (GO:0032502)	1	CDH5	cadherin
Biological Process	immune system process (GO:0002376)	2	IGHA1<br>REG3A	 immunoglobulin<br>-
Biological Process	interspecies interaction between organisms (GO:0044419)	3	IGHA1<br>LYZ<br>REG3A	immunoglobulin<br>-<br>-
Biological Process	localization (GO:0051179)	1	IGHA1	immunoglobulin

Biological Process	metabolic process (GO:0008152)	7	IGHA1<br>CPN1<br>SERPINA7<br>SERPINA5<br>SOD3<br>CNDP1<br>F13A1	immunoglobulin<br>protease<br>protease inhibitor<br>protease inhibitor<br>oxidoreductase<br>microtubule binding motor protein<br>transferase
Biological Process	multicellular organismal process (GO:0032501)	2	F13A1<br>CDH5	transferase<br>cadherin
Biological Process	response to stimulus (GO:0050896)	6	IGHA1<br>SOD3<br>F13A1<br>LYZ<br>REG3A<br>HYOU1	immunoglobulin<br>oxidoreductase<br>transferase<br>-<br>-<br>-
Biological Process	signaling (GO:0023052)	2	IGHA1<br>HYOU1	immunoglobulin<br>-

Molecular Function	binding (GO:0005488)	7	IGHA1<br>SERPINA7<br>SERPINA5<br>SOD3<br>DNAH3<br>REG3A<br>CDH5	immunoglobulin<br>protease inhibitor<br>protease inhibitor<br>oxidoreductase<br>microtubule binding motor protein<br>-<br>cadherin
Molecular Function	catalytic activity (GO:0003824)	10	CPN1<br>SERPINA7<br>SERPINA5<br>SOD3<br>CNDP1<br>LCAT<br>DNAH3<br>F13A1<br>LYZ<br>MASP1	protease<br>protease inhibitor<br>protease inhibitor<br>oxidoreductase<br>metalloprotease<br>acyltransferase<br>microtubule binding motor protein<br>transferase<br>-<br>serine protease
Molecular Function	molecular function regulator (GO:0098772)	2	SERPINA7<br>SERPINA5	protease inhibitor<br>protease inhibitor
Molecular Function	molecular transducer activity (GO:006008	1	REG3A	-

Cellular Component	cellular anatomical entity (GO:0110165)	14	IGHA1<br>CPN1<br>SERPINA7<br>SERPINA5<br>SOD3<br>CNDP1<br>LCAT<br>DNAH3<br>F13A1<br>IGFALS<br>MASP1<br>REG3A<br>HYOU1<br>CDH5	immunoglobulin<br>protease<br>protease inhibitor<br>protease inhibitor<br>oxidoreductase<br>metalloprotease<br>acyltransferase<br>microtubule binding motor protein<br>transferase<br>transmembrane signal receptor<br>serine protease<br>-<br>-<br>cadherin
Cellular Component	intracellular (GO:0005622)	3	CNDP1<br>DNAH3<br>HYOU1	metalloprotease<br>microtubule binding motor protein<br>-
Cellular Component	protein-containing complex (GO:0032991)	4	IGHA1<br>DNAH3<br>HYOU1<br>CDH5	immunoglobulin<br>microtubule binding motor protein<br>-<br>cadherin
